# Simulated microgravity inhibits osteogenic differentiation of mesenchymal stem cells via depolymerizing F-actin to impede TAZ nuclear translocation

**DOI:** 10.1038/srep30322

**Published:** 2016-07-22

**Authors:** Zhe Chen, Qing Luo, Chuanchuan Lin, Dongdong Kuang, Guanbin Song

**Affiliations:** 1 Key Laboratory of Biorheological Science and Technology, Ministry of Education, College of Bioengineering, Chongqing University, Chongqing 400044, China

## Abstract

Microgravity induces observed bone loss in space flight, and reduced osteogenesis of bone mesenchymal stem cells (BMSCs) partly contributes to this phenomenon. Abnormal regulation or functioning of the actin cytoskeleton induced by microgravity may cause the inhibited osteogenesis of BMSCs, but the underlying mechanism remains obscure. In this study, we demonstrated that actin cytoskeletal changes regulate nuclear aggregation of the transcriptional coactivator with PDZ-binding motif (TAZ), which is indispensable for osteogenesis of bone mesenchymal stem cells (BMSCs). Moreover, we utilized a clinostat to model simulated microgravity (SMG) and demonstrated that SMG obviously depolymerized F-actin and hindered TAZ nuclear translocation. Interestingly, stabilizing the actin cytoskeleton induced by Jasplakinolide (Jasp) significantly rescued TAZ nuclear translocation and recovered the osteogenic differentiation of BMSCs in SMG, independently of large tumor suppressor 1(LATS1, an upstream kinase of TAZ). Furthermore, lysophosphatidic acid (LPA) also significantly recovered the osteogenic differentiation of BMSCs in SMG through the F-actin-TAZ pathway. Taken together, we propose that the depolymerized actin cytoskeleton inhibits osteogenic differentiation of BMSCs through impeding nuclear aggregation of TAZ, which provides a novel connection between F-actin cytoskeleton and osteogenesis of BMSCs and has important implications in bone loss caused by microgravity.

Gravity is necessary to maintain biological processes in most tissues throughout the body and plays a crucial role in regulating bone remodeling and homeostasis^1^,^2^. Microgravity during spaceflight induces significant bone loss in weight-bearing bones at a rate of approximately 1 to 2% per month^3^. The reduced bone formation and increased bone resorption play important roles in the process of microgravity-induced bone loss^4^,^5^. Bone marrow mesenchymal stem cells (BMSCs) are important progenitor and supporting cells that act as a major source for osteoblasts. Abundant evidence has confirmed that microgravity inhibits osteogenesis of BMSCs but promotes adipogenesis^6^,^7^. Our previous studies have also demonstrated that simulated microgravity inhibits the expression of a key transcriptional factor for osteogenesis (Runt-related transcription factor 2, Runx2) but promotes the expression of a key transcriptional factor for adipogenesis (peroxisome proliferator activated receptor γ, PPARγ)^8^. Although efforts have been made to illuminate the mechanisms and several signal pathways that play important roles in the reduced osteogenesis of BMSCs under microgravity, the underlying mechanisms remain unclear.

Actin is a main component of cytoskeleton in eukaryotic cells and is formed by individual actin proteins known as globular actin (G-actin) and filamentous actin (F-actin). F-actin filaments consist of multiple G-actin subunits that interact with one another and are constantly assembled and disassembled^9^,^10^. The dynamic remolding of actin cytoskeleton participates in most cellular behaviors, including deformation, proliferation, migration, signal transduction, and apoptosis^11^. Alternation of actin cytoskeleton generates mechanical signals that cells receive and respond to, and actin cytoskeleton forms a structure to transform mechanical forces into biochemical signals. Due to the contractibility of the actin filaments, proteins associated with the actin cytoskeleton may be stretched, which results in unfolding and the presentation of new binding sites. Such mechanisms can lead to an activation of signaling proteins by phosphorylation. In addition, forces within this tense actin cytoskeleton are transmitted to the nucleus through the linker of nucleoskeleton and cytoskeleton (LINC) complex^12^,^13^,^14^. Emerging evidence reveals that abnormal regulation or function of the actin cytoskeleton may cause numerous diseases^15^,^16^. In particular, the actin cytoskeleton of BMSCs exhibits significant redistribution and reorganization in real or simulated microgravity (SMG)^17^,^18^. However, to our knowledge, little is known about the role of the actin cytoskeleton in osteogenic processes of BMSCs in microgravity.

A crucial step forward in understanding this process has been the discovery of the mechanical signals transduced by two related transcriptional coactivators, YAP (Yes-associated protein) and TAZ (transcriptional coactivator with PDZ-binding motif). The transcriptional coactivators shuttle between the cytoplasm and nucleus, where they interact with TEAD (TEA domain family member) transcriptional factors to regulate gene transcription^19^,^20^. Importantly, TAZ translocates into the nucleus and regulates BMSCs differentiation fate by promoting osteogenesis through activating Runx2, which is a key transcriptional factor for osteogenesis^21^,^22^. Several mechanical and chemical stimuli can activate TAZ and promote osteogenesis of BMSCs, whereas the role of the actin cytoskeleton in this process is not well understood^23^,^24^. LATS1 (large tumor suppressor 1) is a novel actin-binding protein, and F-actin depolymerization can activate LATS1, which inhibits TAZ nuclear translocation through phosphorylation^25^,^26^. However, whether the actin cytoskeleton regulates TAZ nuclear aggregation through LATS remains controversial^26^,^27^. A better understanding of the connection between the actin cytoskeleton and TAZ activation in the osteogenic process of BMSCs may provide novel insights into the mechanisms of bone loss during space flight.

In the present study, we investigated the influence of SMG on TAZ nuclear aggregation in rat BMSCs and the role of F-actin reorganization in this process. Our data indicated that F-actin reorganization mediates the inhibited osteogenic differentiation of BMSCs through impeding TAZ nuclear translocation in SMG and that stabilization of the actin cytoskeleton induced by Jasp (Jasplakinolide) or LPA (lysophosphatidic acid) could be an efficient intervention to retain the osteogenic differentiation of BMSCs.

## Results

### Remodeling of F-actin affects osteogenic differentiation of BMSCs

**Figure 1:**
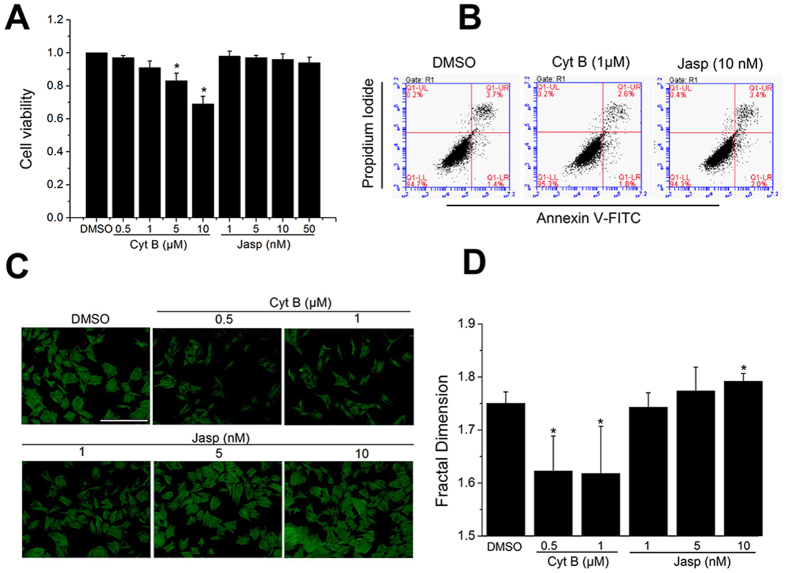
The effect of actin-perturbing drugs on cell toxicity and F-actin organization. (**A**) MTT analysis of BMSCs after treatment with Cyt B or Jasp for 48 h. For controls, BMSCs were cultured in medium containing 0.1% DMSO. (n = 3). (**B**) Cell apoptosis was determined by Annexin V/PI staining. BMSCs were treated with Cyt B or Jasp for 48 h. (n = 3). (**C**) Fluorescence images of the actin cytoskeleton (green) cultured for 48 h in osteogenic differentiation medium containing drugs at the indicated concentrations. (**D**) Fractal dimension analysis of morphological changes in F-actin cytoskeleton after 48 h of treatment with Cyt B or Jasp at the indicated concentrations (total 50 cells, n = 3). For each group, values are mean ± SD. *p < 0.05 vs. the DMSO group.

To evaluate the effect of F-actin reorganization on osteogenesis and TAZ activation of BMSCs, we treated BMSCs with varying levels of Cytochalasin B (Cyt B) and Jasplakinolide (Jasp), which can directly depolymerize and polymerize F-actin, respectively. The MTT results revealed that a high dosage of Cyt B (5 μM and 10 μM) repressed cell viability. The cell viability of BMSCs treated with Jasp (up to 50 nM) exhibited slight changes compared with the control group (Fig. 1A). The flow cytometry results confirmed that the treatment of BMSCs with Cyt B (1 μM) or Jasp (10 nM) for 48 h had no significant impact on cell apoptosis (Fig. 1B). The actin staining results indicated that Cyt B obviously leads to a dose-dependent F-actin depolymerization, whereas Jasp dramatically promoted the polymerization of F-actin (Fig. 1C). Fractal geometry provides a means for quantitatively analyzing structures in terms of their fractal dimension (D), a statistic describing the degree of complexity of the structure^27^. Here, we utilized fractal box count analysis to reveal the changes in the D value and found that Cyt B significantly decreased the D value of F-actin, but Jasp (10 nM) induced an increase in the D value (Fig. 1D).

**Figure 2:**
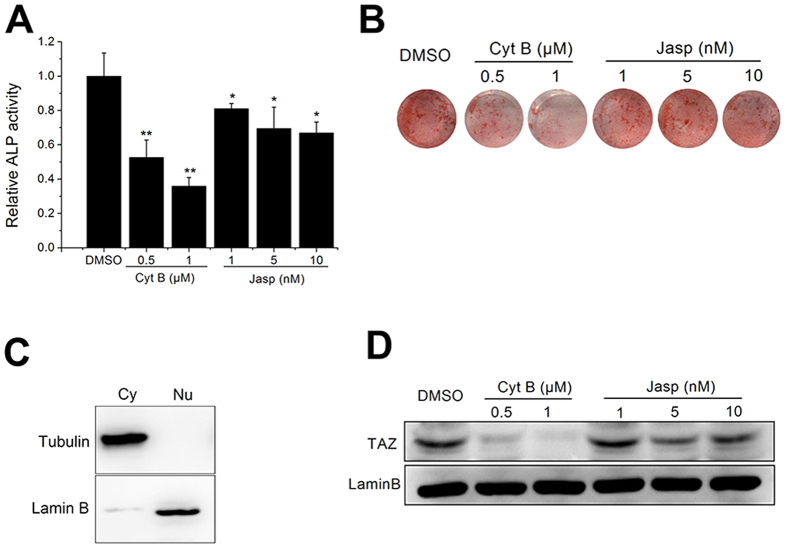
The effect of Cyt B and Jasp on TAZ nuclear aggregation and osteogenesis in BMSCs. (**A**) ALP activity analysis of BMSCs after treatment with Cyt B or Jasp for 7 days. (n = 3). (**B**) AR-S of BMSCs after treatment with Cyt B or Jasp for 14 days. (n = 3). (**C**) Western blot analysis for the expression of tubulin and Laminin B in the cytoplasmic (Cy) and nuclear (Nu) fractions. (n = 3). (**D**) Western blot analysis of the relative nuclear translocation of TAZ after exposure to actin-perturbing drugs for 48 h. Lamin B was used as loading control for nuclear extracts. (n = 3). For each group, values are mean ± SD. *p < 0.05, **p < 0.01 vs. the DMSO group.

Next, we sought to explore the influence of F-actin assembly on the osteogenic differentiation of BMSCs. ALP (alkaline phosphatase) activity assay results revealed that polymerization or depolymerization of F-actin inhibited ALP activity at day 7, and the effects on depolymerization were more obvious (Fig. 2A). AR-S (alizarin red staining) results also showed that depolymerization of F-actin severely repressed the calcium deposition of BMSCs at day 14, but the calcium deposition of BMSCs treated with Jasp was similar to the control group (Fig. 2B). Although Jasp facilitated polymerization of actin cytoskeleton and repressed ALP activity, no obvious influence on calcium deposition was noted, which revealed the terminal osteogenic differentiation of BMSCs. To evaluate the impact of F-actin assembly on TAZ activation of BMSCs, we isolated cytoplasmic and nuclear proteins from BMSCs, and the western blot results demonstrated the efficiency of nuclear protein isolation (Fig. 2C). Then, we found that nuclear aggregation of TAZ was dramatically inhibited by Cyt B, but Jasp had no obvious effect on TAZ nuclear translocation (Fig. 2D). These results demonstrated that depolymerized F-actin obviously inhibits both osteogenic differentiation and TAZ nuclear translocation in BMSCs.

**Figure 3:**
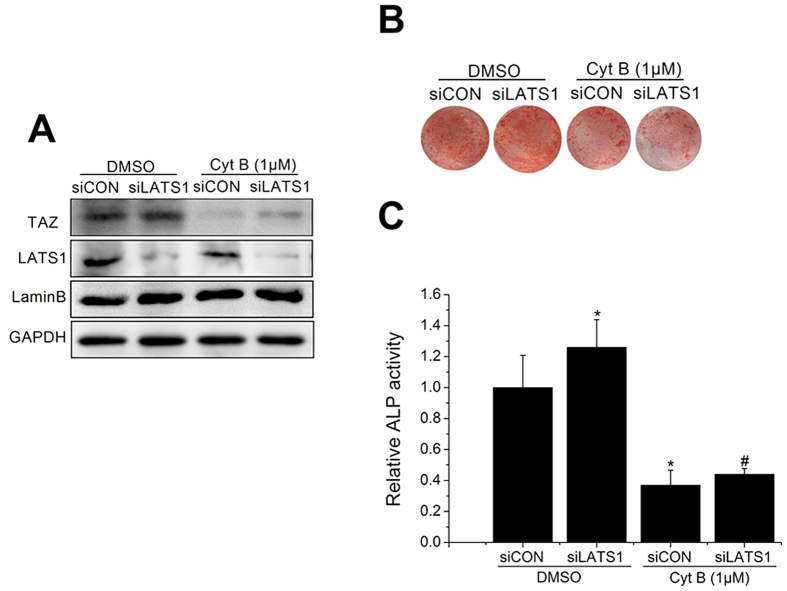
Depolymerized F-actin inhibited TAZ activation and osteogenesis in BMSCs independently of LATS. (**A**) Western blot analysis of TAZ nuclear aggregation and total LATS1 expression in BMSCs transfected with siCON or siLATS1 with or without Cyt B after 48 h. Lamin B and GAPDH were used as loading controls for nuclear extracts and cell extracts, respectively. (n = 3). (**B**) AR-S of BMSCs transfected with siCON or siLATS1 after 14 days. (n = 3). (**C**) ALP activity analysis for BMSCs transfected with siCON or siLATS1 at day 7. (n = 3). For each group, values are mean ± SD. *p < 0.05 vs. the DMSO + siCON group, #*p* < 0.05 vs. the DMSO + siLATS1 group.

To evaluate the role of LATS1 in decreased TAZ nuclear aggregation and reduced osteogenic differentiation of BMSCs caused by Cyt B, we transfected BMSCs with siLATS1 or siCON, and western blot analysis results suggested that siLATS1 transfection efficiently decreased LATS1 expression compared with the siCON group (Fig. 3A). Depletion of LATS1 increased the nuclear aggregation of TAZ. Conversely, TAZ nuclear location was not recovered by siLATS1 transfection under treatment with Cyt B (Fig. 3A). AR-S and ALP activity assay results further verified that depletion of LATS1 could not rescue the osteogenic differentiation of BMSCs inhibited by Cyt B (Fig. 3B,C), indicating that depolymerization of F-actin impedes TAZ nuclear aggregation and osteogenesis in BMSCs independently of LATS.

### SMG disturbs F-actin and inhibits TAZ nuclear translocation

**Figure 4:**
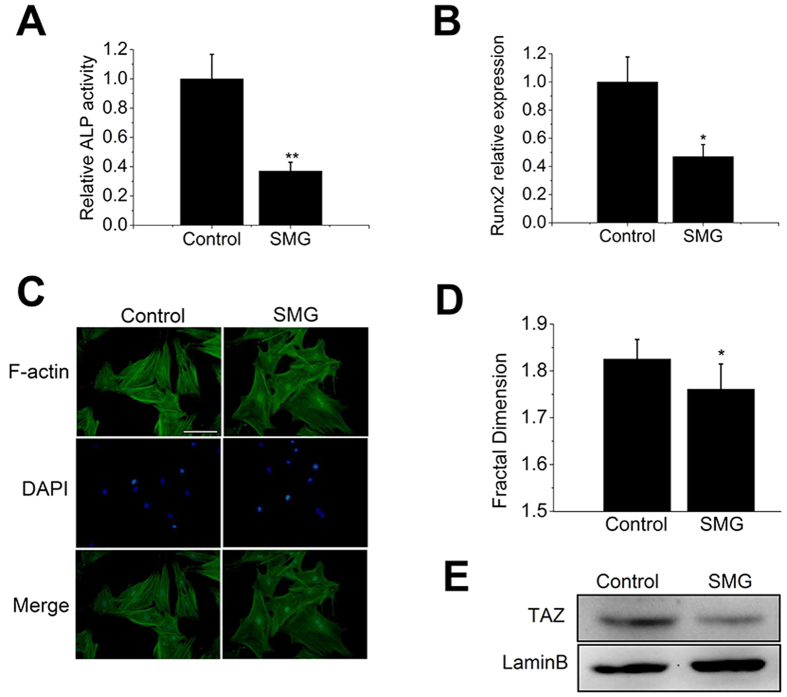
SMG inhibited osteogenic differentiation of BMSCs. (**A**) ALP activity analysis of BMSCs after exposure to SMG for 48 h cultured in osteogenic medium. (n = 3). (**B**) Real-time PCR of relative Runx2 mRNA levels in BMSCs after exposure to SMG for 48 h. (n = 3). (**C**) Fluorescence images of the actin cytoskeleton (green) and nucleus (blue) of BMSCs cultured for 48 h in SMG. (**D**) Fractal analysis of morphological changes in F-actin cytoskeleton (total of 50 cells, n = 4). (**E**) Western blot analysis of TAZ nuclear aggregation in BMSCs. Lamin B was used as a loading control for nuclear extracts. (n = 4). For each group, values are mean ± SD. *p < 0.05, **p < 0.01 vs. the Control group.

To study the impact of SMG on osteogenic differentiation of BMSCs, we utilized a clinostat to simulate the microgravity effect. It has been reported that the early 48 hours are vital for osteogenic differentiation of BMSCs and that actin responds to microgravity rapidly^3^,^18^. The ALP activity assay and real-time PCR results indicated that SMG seriously inhibited the ALP activities of BMSCs after exposure to SMG for 48 h (Fig. 4A) and reduced the expression of Runx2, which acts as a key transcriptional factor for osteogenesis (Fig. 4B). To determine whether the actin cytoskeleton of BMSCs was disturbed in SMG, we examined filamentous F-actin using phalloidin. SMG induced significant reorganization of F-actin, which became thinner and disordered and exhibited a dispersed distribution (Fig. 4C). Fractal analysis revealed that SMG dramatically decreased the D value of the actin cytoskeleton (Fig. 4D). To further evaluate TAZ activation in BMSCs in SMG, we isolated nuclear proteins and found that SMG obviously repressed TAZ nuclear aggregation (Fig. 4E). These results demonstrated that SMG inhibits both osteogenic differentiation and TAZ activation in BMSCs.

### Jasp recovers osteogenic differentiation of BMSCs in SMG

**Figure 5:**
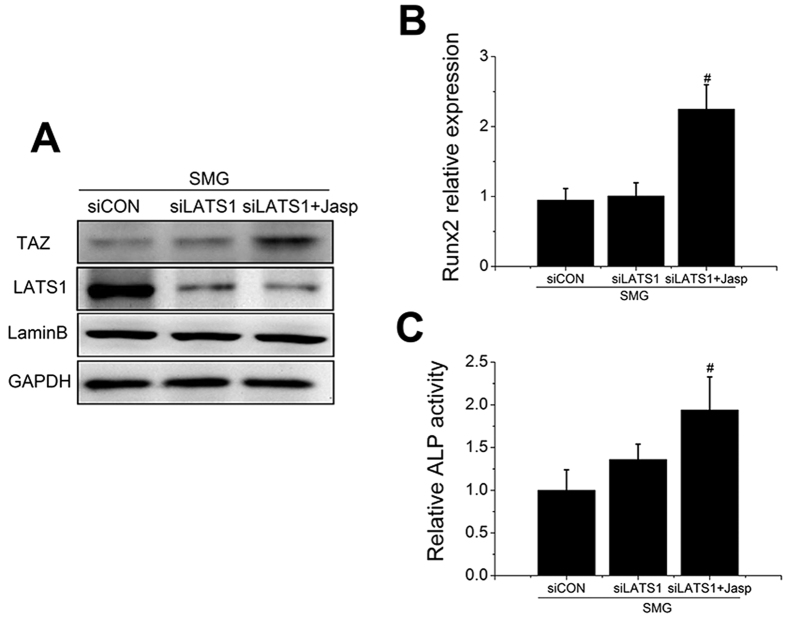
Jasp recovered osteogenic differentiation of BMSCs in SMG. (**A**) Western blot analysis for the nuclear aggregation of TAZ and total LATS1 expression in BMSCs transfected with siCON or siLATS1 and cultured in osteogenic medium with or without Jasp (10 nM) after 48 h. Lamin B and GAPDH were used as loading controls for nuclear extracts and cellular extracts, respectively. (n = 3). (**B**) ALP activity analysis of BMSCs after exposure to SMG for 48 h. (n = 3). (**C**) Real-time RT-PCR of relative Runx2 mRNA levels in BMSCs after exposure to SMG for 48 h. (n = 3). For each group, values are mean ± SD. #*p* < 0.05 vs. siLATS1 + SMG group.

To evaluate the role of LATS in reduced TAZ nuclear translocation and decreased osteogenic differentiation of BMSCs in SMG, we transfected BMSCs with siLAT1 or siCON, and western blot results confirmed the efficiency of siRNA (Fig. 5A). However, no obvious increase in TAZ nuclear location was noted in SMG. In addition, depletion of LATS1 could not rescue ALP activity and the expression of Runx2 inhibited by SMG (Fig. 5B,C). Conversely, we found that Jasp (10 nM) significantly increased the nuclear aggregation of TAZ under conditions of LATS1 deletion. Moreover, Jasp also obviously promoted ALP activity and Runx2 expression in BMSCs in SMG (Fig. 5B,C). These results suggested that SMG may inhibit osteogenic differentiation of BMSCs through the F-actin-TAZ pathway independently of LATS.

### LPA rescues osteogenic differentiation of BMSCs in SMG via the F-actin-TAZ pathway

**Figure 6:**
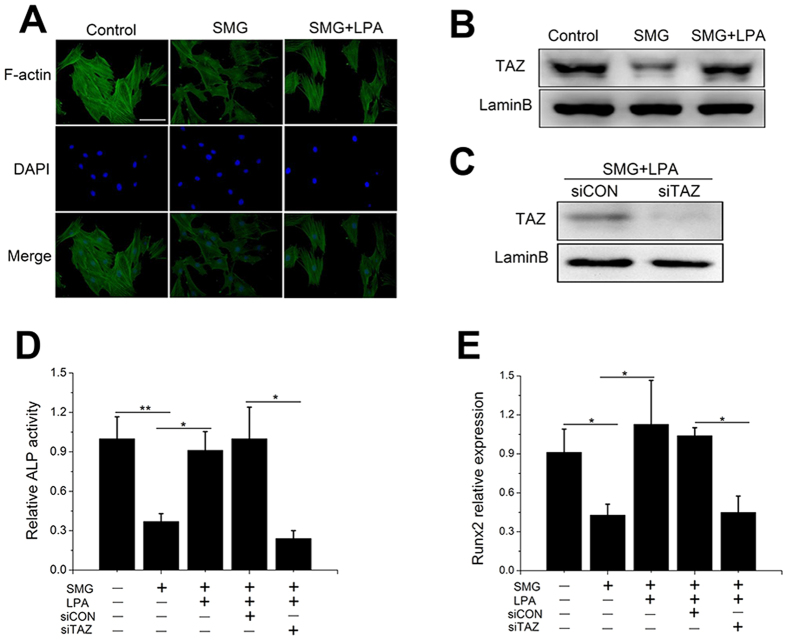
LPA rescued osteogenic differentiation of BMSCs through the TAZ pathway. (**A**) Fluorescence images of the actin cytoskeleton (green) and nucleus (blue) of BMSCs cultured for 48 h in SMG with or without LPA (2 μM). (n = 4). (**B**) Western blot analysis of TAZ nuclear aggregation in BMSCs after exposure to SMG for 48 h. Lamin B was used as a loading control for nuclear extracts. (n = 3). (**C**) Western blot analysis of TAZ nuclear aggregation in BMSCs transfected with siCON or siTAZ after exposure to SMG for 48 h cultured in osteogenic medium. (n = 3). (**D**) ALP activity analysis of BMSCs after exposure to SMG for 48 h. (n = 3). (**E**) Real-time RT-PCR of relative Runx2 mRNA levels in BMSCs after exposure to SMG for 48 h. (n = 3). For each group, values are mean ± SD. *p < 0.05, **p < 0.01.

To confirm the hypothesis that SMG inhibits osteogenic differentiation of BMSCs via depolymerized F-actin, we attempted to stabilize F-actin through the RhoA (Ras homolog gene family, member A) pathway in SMG. LPA is a bioactive lysophospholipid involved in numerous physiological responses and promotes RhoA activation through the LPA 1 receptor^28^. Indeed, actin staining revealed that BMSCs treated with LPA exhibited a polymerized and thicker actin network (Fig. 6A). Importantly, LPA rescued TAZ nuclear location in BMSCs in SMG (Fig. 6B). Moreover, LPA recovered ALP activity and Runx2 expression in BMSCs in SMG (Fig. 6D,E). To further evaluate the role of TAZ in the increased osteogenic differentiation of BMSCs induced by LPA in SMG, we utilized siRNA to deplete TAZ, and western blot results showed that siTAZ transfection dramatically decreased TAZ nuclear aggregation compared with the siCON group (Fig. 6C). Crucially, depletion of TAZ obviously inhibited the increased osteogenic differentiation of BMSCs induced by LPA in SMG, which was verified by the ALP activity assay and Runx2 expression (Fig. 6D,E).

**Figure 7:**
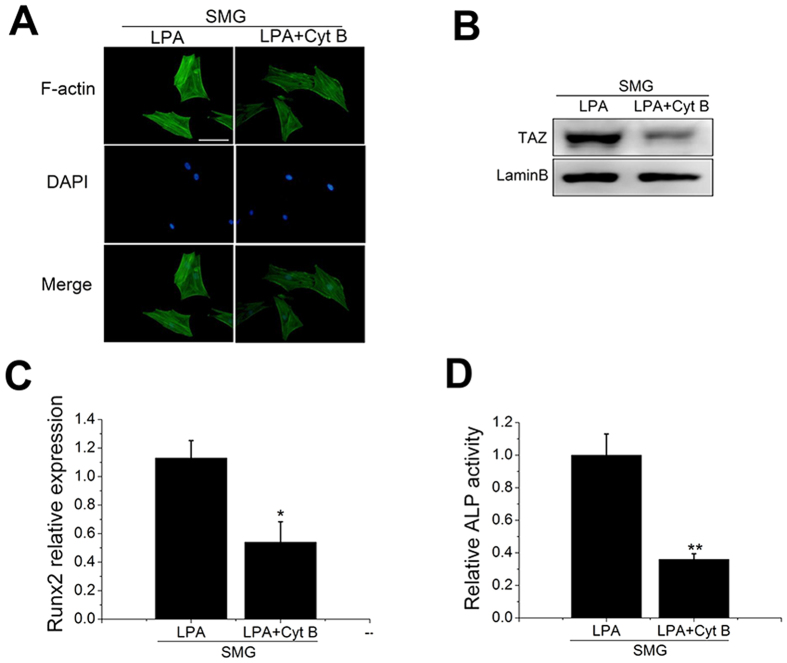
LPA recovered osteogenic differentiation of BMSCs in SMG via F-actin. (**A**) Fluorescence images of the actin cytoskeleton (green) and nucleus (blue) of BMSCs cultured for 48 h in SMG with or without Cyt B (1 μM). (n = 3). (**B**) Western blot analysis of TAZ nuclear aggregation in BMSCs after exposure to SMG for 48 h. Lamin B was used as a loading control for nuclear extracts. (n = 3). (**C**) ALP activity analysis of BMSCs after exposure to SMG for 48 h. (n = 3). (**D**) Real-time RT-PCR of relative Runx2 mRNA levels in BMSC after exposure to SMG for 48 h. (n = 3). For each group, values are mean ± SD. *p < 0.05, **p < 0.01 vs. the SMG + LPA group.

To explore the role of F-actin in TAZ activation and increased osteogenic differentiation of BMSCs induced by LPA in SMG, we treated BMSCs with Cyt B and found that F-actin was depolymerized (Fig. 7A). Importantly, the western blot results indicated that Cyt B notably abrogated the nuclear aggregation of TAZ induced by LPA in SMG (Fig. 7B). Moreover, there was an obvious decline in ALP activity and Runx2 expression in SMG upon treatment with Cyt B (Fig. 7C,D). Thus, these results indicated that LPA rescues osteogenic differentiation of BMSCs primarily via the F-actin-TAZ pathway.

## Discussion

The osteogenic differentiation of BMSCs is sensitive to actin dynamics, but the underlying mechanisms have not been clarified. Here, we established a link among actin reorganization, TAZ nuclear location, and BMSCs osteogenic differentiation. We proposed that SMG inhibits osteogenic differentiation of BMSCs via depolymerizing F-actin to impede TAZ nuclear translocation. Actin plays a vital role in cell morphology maintenance and extracellular signal transduction^28^,^29^. Expression of α-smooth muscle actin promotes osteogenic differentiation but limits clonogenicity and adipogenesis of MSCs through controlling YAP/TAZ activation^30^. Numerous MSCs genes showed significant changes induced by latrunculin B (an actin-perturbing drug)^31^. Crucially, several key transcriptional factors or co-activator factors are regulated by the reorganization of actin, such as p53, NKX2.5, and TAZ^31^,^32^,^33^. One of the mechanisms by which actin regulates the activation of the above proteins involves affecting its distribution between the cytoplasm and nucleus. The polymerization of actin impaired the nuclear import of p53 by anchoring p53 on polymerized actin in the cytoplasm^32^. Nevertheless, the nuclear translocation of TAZ relies on a polymerized actin structure^34^. F-actin depolymerization activates LATS and thereby phosphorylates TAZ, preventing TAZ nuclear location^35^,^36^. However, TAZ has been identified as a mechanical sensor and is regulated by the actin structure independently of LATS^26^. Moreover, LATS of human embryonic stem cells phosphorylates YAP/TAZ but does not impede its nuclear location^37^. Hence, the interplay among actin, TAZ, and osteogenesis is complex and not entirely understood at present.

Here, we utilized Cyt B and Jasp to remodel F-actin, which directly polymerize and depolymerize F-actin, respectively. F-actin depolymerization obviously repressed the ALP activities and calcium deposition of BMSCs (Fig. 2A,B). The actin-depolymerizing drugs cytochalasin D and latrunculin A inhibit ALP in human MSCs^38^. These results confirmed that F-actin plays an important role in the osteogenesis of BMSCs and that the osteogenic process of BMSCs requires polymerized F-actin. We also observed reduced TAZ nuclear aggregation in BMSCs with depolymerized F-actin induced by Cyt B (Fig. 2D). However, depletion of LATS1 could not rescue TAZ nuclear localization and the osteogenesis of BMSCs inhibited by Cyt B (Fig. 3). Thus, it appears that depolymerized F-actin impeded TAZ translocation into the nucleus independently of LATS1, suggesting that LATS1 is not a main determinant in this process. The mechanisms of actin regulating TAZ nuclear location via a LATS-independent pathway remain unclear. The loss of the cytoskeletal to nuclear connectivity through the knockdown of nesprin-1 reduced the YAP/TAZ response to dynamic strectch^39^. Thus, it is possible that F-actin reorganization affects the structure and function of the nucleus via the linker of nucleoskeleton and cytoskeleton, resulting in inhibition of TAZ nuclear translocation. Our study first revealed the interplay among F-actin, TAZ, and LATS1 in the process of osteogenesis.

Previous studies have revealed that microgravity inhibits the osteogenic differentiation of BMSCs but promotes adipogenic differentiation^40^,^41^. Moreover, microgravity disturbs actin organization and cell function^42^,^43^. However, the role of actin dynamics induced by microgravity in osteogenic differentiation of BMSCs remains obscure. Here, we utilized a clinostat to model SMG and confirmed the inhibitory effect of SMG on osteogenic differentiation of BMSCs (Fig. 4A,B). We also detected that SMG obviously induced the depolymerization of F-actin and inhibited TAZ nuclear translocation (40%, data not shown) (Fig. 4C,E). Given that TAZ is necessary for BMSC osteogenesis, we hypothesized that SMG inhibits osteogenic differentiation of BMSCs via depolymerizing actin and thereby impeding TAZ nuclear location. Looking for effective interventions to retain BMSCs osteogenic potential in SMG has garnered significant attention^44^,^45^. The over-expression of constitutively active RhoA or the combination of BMP (bone morphogenetic protein), fibroblast growth factor 2, and p38 inhibitor could, to some extent, increase osteogenic differentiation and repress the adipogenic differentiation of hBMSCs in SMG^46^,^47^. Human MSCs treated with Phalloidin (a drug that stabilizes polymerized actin) or siRNA targeted at two main actin-depolymerizing factors (confilin1 and destrin) exhibited increased osteogenesis^48^. Here, we attempted to rescue osteogenic differentiation of BMSCs under SMG through stabilization of actin. Jasp directly binds to actin and induces actin polymerization and stabilization^49^, whereas LPA is a pluripotent lipid mediator that is present at high concentrations in blood serum and has been recognized as an activator of RhoA^50^. Activated RhoA promotes actin polymerization through the Rock-confilin pathway^51^. Similarly, we demonstrated that LPA stabilized F-actin under SMG (Fig. 6A). Interestingly, both Jasp and LPA obviously increased TAZ nuclear location and promoted osteogenic differentiation of BMSCs under SMG (Figs 5 and 6). Treatment with siTAZ and Cyt B confirmed that LPA mainly acts through the F-actin-TAZ pathway. Hence, the strategy of stabilizing F-actin to rescue BMSC osteogenic differentiation under SMG through the TAZ pathway could be effective. Further study is required to detect the response of actin to microgravity *in vivo* and explore the possibility of LPA as an intervention drug for bone loss caused by microgravity.

## Conclusion

In summary, our data suggest that the scale of TAZ activation may be broad and regulated by F-actin and LATS. In particular, the osteogenic process of BMSCs requires a polymerized actin cytoskeleton to facilitate TAZ nuclear translocation. Simulated microgravity inhibits osteogenic differentiation of mesenchymal stem cells via depolymerizing F-actin to impede TAZ nuclear translocation. Our findings provide new insights into the connection between the osteogenesis of BMSCs and actin cytoskeleton and propose potential mechanisms leading to reduced bone mass under microgravity.

## Materials and Methods

### Cell isolation and treatments

All animal experiments in this study were approved by the Local Committee of Animal Use and Protection of Chongqing University. The methods were performed in accordance with the approved guidelines. Rat BMSCs were isolated by the Percoll density gradient centrifugation method as described previously^52^. Briefly, the femur and tibia from Sprague-Dawley rats were sawed, and gelatinous marrow was extracted under sterile conditions. BMSCs were separated by density gradient centrifugation using Percoll (Sigma, USA d = 1.037 g/mL) for 30 min at 2500 rpm. Then, BMSCs were enriched in the intermediate zone and cultured in Dulbecco’s modified Eagle’s medium (DEME, Hyclone, USA) with 10% fetal bovine serum (FBS, Gibco, USA), penicillin (100 U/mL), and streptomycin (100 μg/mL) at 37 °C with 5% CO_2_. In this study, all of the cells used were between passages 3 and 5. For osteogenic differentiation, in confluent conditions, the culture medium was changed to the differentiation medium (DEME containing 10% FBS, 100 nM dexamethasone [Sigma, USA], 10 mM β-glycerol phosphate [Sigma, USA], and 50 μg/mL ascorbic acid [Sigma, USA]). To perturb the actin cytoskeleton, the following drugs were used in the experiments: Cytochalasin B (Cyt B) (0.5 μM, 1 μM), (Aladdin, China) and Jasplakinolide (Jasp) (1 nM, 5 nM, 10 nM) (Santa Cruz, USA). For RhoA activation, 2 μM lysophosphatidic acid (LPA [Sigma, USA]) was added to the medium.

### Clinorotation to simulate microgravity

The 2D-clinostat device utilized in this study was constructed by the National Microgravity Laboratory, Institute of Mechanics, Chinese Academy of Sciences, China. Clinostats are an effective ground-based tool to stimulate certain effects under microgravity on the principle that continuous rotation constantly changes cell orientation with respect to gravity^53^,^54^. The clinostat used in this experiment is a device that provides a vector-averaged reduction in the apparent gravity on cells, and the average gravitational force acting on the cells generated by the clinostat is reduced to approximately 10^−3^ g when the clinostat rotates at 10 rpm compared with normal gravity (1 g). Details and application of the method were previously described^8^. Briefly, BMSCs were injected into a chamber at a total of 3 × 10^5^ cells, and all air bubbles were carefully removed to prevent fluid shear stress. After cells attached to the slide for 24 h, the chambers were fixed at the clinostat, and the effect of simulated microgravity was achieved by rotation around the horizontal axis at 10 rpm for 48 h in an incubator at 37 °C. As controls, static cells were cultured in a chamber under the same conditions but were not subjected to rotation.

### MTT assay

To assay cell viability, BMSCs were seeded at a density of 5000 cells/cm^2^ in 24-well plates. After adherence, BMSCs were treated with Cyt B or Jasp for 48 h. Then, cells were incubated with a 2 mg/mL solution of MTT for 4 h at 37 °C. Then, the supernatant was removed, and 600 μL DMSO (dimethyl sulfoxide) was applied to the cells for 15 min. Finally, 100 μL of the supernatant was collected and transferred to a 96-well plate. Absorbance was measured at 540 nm using a microplate reader (BIO-RAD, USA).

### Apoptosis analysis by flow cytometry

Cells were harvested, washed and stained with Annexin V-FITC and PI for 15 min in the dark at room temperature. Cells stained with Annexin V-FITC and PI were acquired on an LSRII (BD, USA). The percentages of viable cells, necrotic cells, and early and late apoptotic cells were compared.

### Real time-PCR

Total cellular RNA was isolated using an RNA extraction kit (Bioteke, Beijing, China) according to the manufacturer’s instructions. After the reverse transcription reaction, real-time PCR was performed using the ABI 7500 system and SYBR Premix Ex Taq (Bioteke, Bejing, China) according to the manufacturer’s instructions. The following real-time PCR conditions were employed: 40 cycles at 94 °C for 5 s and 58 °C for 30 s. The dissociation stage was added to the end of the amplification procedure. Nonspecific amplification was not detected using the dissociated curve. GAPDH was used as an internal control. Sense and antisense primers were as follows:

Runx2:

5′-CAGACCAGCAGCACTCCATA-3′ and 5′-CAGCGTCAACACCATCATTC-3′

GAPDH:

5′-GGCACAGTCAAGGCTGAGAATG-3′ and 5′-ATGGTGGTGAAGACGCCAGTA-3′.

### Western-blot analysis

BMSCs whole cell lysates were collected using cell lysis buffer (62 mM Tris/HCl, pH 6.8, 10% v/v glycerol, 2% v/v SDS, 2% V/V β-mercaptoethanol, and 0.02% Bromophenol Blue) supplemented with a protease inhibitor cocktail (Roche, USA) and phosphatase inhibitor cocktail I and II (Sigma-Aldrich, USA). BMSC nuclear extracts were collected using NXTRACT kit (Bioteke, China) per the manufacturer’s protocol. Equal protein aliquots (30 μg) were separated by 10% v/v SDS-PAGE, and proteins were electroblotted onto PVDF membranes. The membranes were blocked with Tri-buffered saline containing 0.1% Tween 20 (TBST) and 5% v/v bovine serum albumin for 1 h at room temperature. Antibodies against TAZ (Cell signaling, USA), LATS1 (Santa Cruz, USA), tubulin (Beyotime, China), Lamin B (BOSTER, China) and GAPDH (CWBIO, China) were used according to the manufacturers’ protocols. Incubation was performed overnight at 4 °C with gentle shaking. Then, the membrane was incubated with an HRP-conjugated antibody (goat anti rabbit IgG, ZSGB-BIO, China) for 1 h at room temperature. Protein expression was visualized using an enhanced electrogenerated chemiluminescence (ECL) system (VersaDoc, Bio-Rad, USA).

### Actin staining and Fractal analysis

Cells were fixed with 4% paraformaldehyde for 30 min and preincubated in blocking buffer (1% BSA/PBS) for 30 min. Cells were then incubated in Alexa 488-conjugated phalloidin (5 μg/mL, Molecular Probes, USA) in blocking buffer at room temperature for 2 h, briefly washed three times in PBS, and mounted in Mowiol. Images were captured by an Eclipse 80i Fluorescence microscope (Nikon). The fractal box count analysis of ImageJ software was used to analyze the fractal dimension (D) of actin cytoskeleton according to Fuseler’s report^55^.

### Small interfering RNA transfection

Small interfering RNA (siRNA) duplex oligos targeting TAZ and LATS1 mRNA (siTAZ and siLATS1) and non-targeting duplex oligos, which served as a negative control (siCON), were synthesized by Shanghai Genepharma Corporation, China. After seeding for 24 h, cells were transfected with 2 μg/ml of siRNA using RNAi-Mate (Shanghai Genepharma Corporation, China) as recommended by the manufacturers’ instructions and cultured in osteogenic medium. Given that the calcium depositions of BMSCs lasted for 14 days, cells were subjected to two rounds of transfection prior to performing the experiments. The siRNA sequences are listed below:

siTAZ: 5′-GGCCAGAGAUAUUUCCUUATT-3′, 5′-UAAGGAAAUAUCUCUGGCCTT-3′;

siLATS1: 5′-GUCGAUACGAAAGCUUUGUTT-3′, 5′-ACAAAGCUUUCGUAUCGACTT-3′;

siCON: 5′-UUCUCCGAACGUGUCACGTT-3′, 5′-ACGUGACACGUUCGGAGATT-3′.

### Alkaline phosphatase activity assay and alizarin red staining

For quantitative assessment of alkaline phosphatase (ALP) activity, BMSCs were lysed using RIPA lysis buffer (Beyotime), and the cell supernatant was collected in a 96-well plate. Next, substrates and p-nitrophenol from the Alkaline Phosphatase Assay Kit (Beyotime, China) were subsequently added and incubated for 10 min at 37 °C. ALP activity was determined at the wavelength of 405 nm. The results were normalized to the total intracellular protein content determined by the bicinchoninic acid (BCA) Protein Assay Kit.

For alizarin red staining (AR-S), after osteogenic induction for 14 days, BMSCs on the 12-well plate were washed twice with PBS and fixed with 4% paraformaldehyde at room temperature for 10 min. The cells were washed three times with distilled water and incubated with 0.1% AR (Sigma, USA) at 37 °C for 30 min. Cells were then washed thoroughly with distilled water, and the images were acquired using a scanner.

### Statistical analyses

All values are expressed as the mean ± standard deviation (SD). A minimum of three independent experiments was performed for each assay. Statistical analysis was performed using a two-sided unpaired Student’s t-test between two groups. For multiple groups, the statistical significance of two groups was calculated using the Bonferroni *post hoc* test. A level of *p* < 0.05 was accepted as statistically significant.

## Additional Information

**How to cite this article**: Chen, Z. *et al*. Simulated microgravity inhibits osteogenic differentiation of mesenchymal stem cells via depolymerizing F-actin to impede TAZ nuclear translocation. *Sci. Rep.* **6**, 30322; doi: 10.1038/srep30322 (2016).
